# Mitochondrial dysfunction in neurodegenerative diseases and the potential countermeasure

**DOI:** 10.1111/cns.13116

**Published:** 2019-03-19

**Authors:** Yan Wang, Erin Xu, Phillip R. Musich, Fang Lin

**Affiliations:** ^1^ Department of Pharmacology, Laboratory of Aging and Nervous Diseases School of Pharmaceutical Science Soochow University Suzhou China; ^2^ Department of Pediatrics University of North Carolina at Chapel Hill Chapel Hill North Carolina; ^3^ Department of Biomedical Sciences Quillen College of Medicine, East Tennessee State University Johnson City Tennessee

**Keywords:** aging, mitochondria, mitochondrial biogenesis, mitophagy, neurodegenerative diseases

## Abstract

Mitochondria not only supply the energy for cell function, but also take part in cell signaling. This review describes the dysfunctions of mitochondria in aging and neurodegenerative diseases, and the signaling pathways leading to mitochondrial biogenesis (including PGC‐1 family proteins, SIRT1, AMPK) and mitophagy (parkin‐Pink1 pathway). Understanding the regulation of these mitochondrial pathways may be beneficial in finding pharmacological approaches or lifestyle changes (caloric restrict or exercise) to modulate mitochondrial biogenesis and/or to activate mitophagy for the removal of damaged mitochondria, thus reducing the onset and/or severity of neurodegenerative diseases.

## 1 MITOCHONDRIA IN THE NORMAL CONDITION

The mitochondrion is a double‐membrane‐bound organelle in most eukaryotic cell. Generally, mitochondria are between 0.75 and 3 μm in diameter but differ in lengths and structure. Hundreds of mitochondria form cable‐like structures and supply the energy demand of cells through the oxidative phosphorylation (OXPHOS) process.

**Figure 1:**
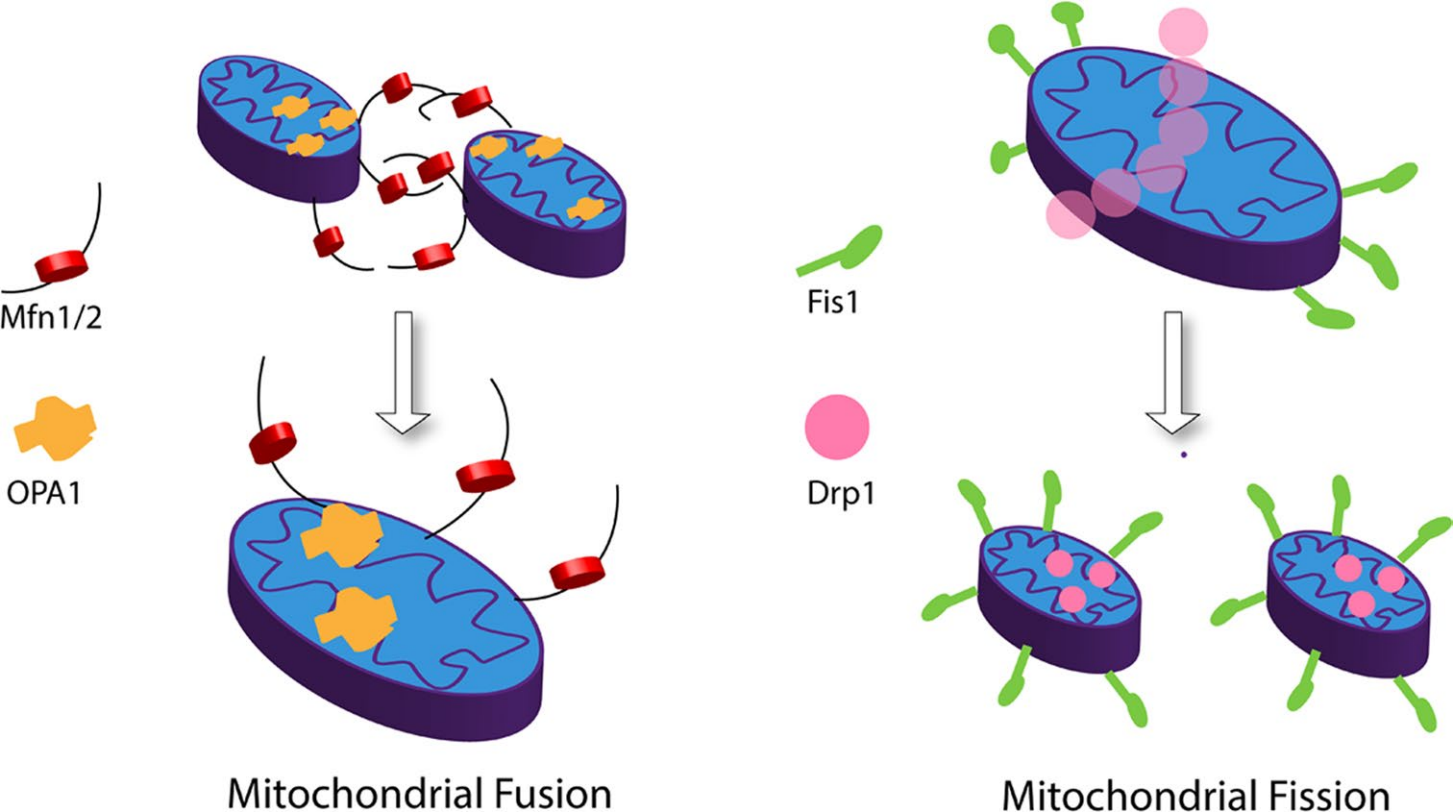
General features of mitochondria fusion and fission. Left: Outer mitochondrial membrane proteins Mfn1 and Mfn2 mediate fusion of the outer mitochondrial membrane while the inner membrane protein OPA1 regulates the fusion of the inner mitochondrial membranes. Right: Fis1 and Drp1 contribute to mitochondrial fission process

Mitochondria constantly divide, fuse, and alter their size and shape, forming a dynamic network to maintain their integrity and quantity. Normal mammalian cells maintain a balance between fusion and fission. Mitofusin 1 and 2 (Mfn1/2) proteins are involved in fusion of the outer mitochondrial membrane, and optic atrophy 1 (OPA1) protein mediates fusion of the inner mitochondrial membranes. Mitochondrial fission 1 (Fis1) and dynamin‐related protein 1 (Drp1) are associated with mitochondrial fission. Fis1 localizes primarily on the outer mitochondrial membrane. Drp1is a cytoplasmic protein that translocates to mitochondria and interacts with Fis1 to enhance fission.^1^ Figure 1 illustrates these general features of mitochondrial fission and fusion.

A positive mitochondrial membrane potential (Δψm) of 120‐200 mV is fundamental for the normal performance and survival of cells, especially those that have a high‐energy requirement. Thus, loss of Δψm is an indicator of reduced cell health. The collapse of Δψm due to the opening of a high‐conductance pore in the inner mitochondrial membrane is part of the molecular mechanism in apoptosis. The mitochondrial uncoupler carbonyl cyanide‐m‐chlorophenylhydrazone (CCCP) depolarizes the inner mitochondrial membrane, reducing Δψm and ATP production, thus increasing the level of AMP and the phosphorylated (active) AMP‐activated protein kinase (pAMPK). This sequence of events elevates the level of reactive oxygen species (ROS), leading to oxidative damage.

**Figure 2:**
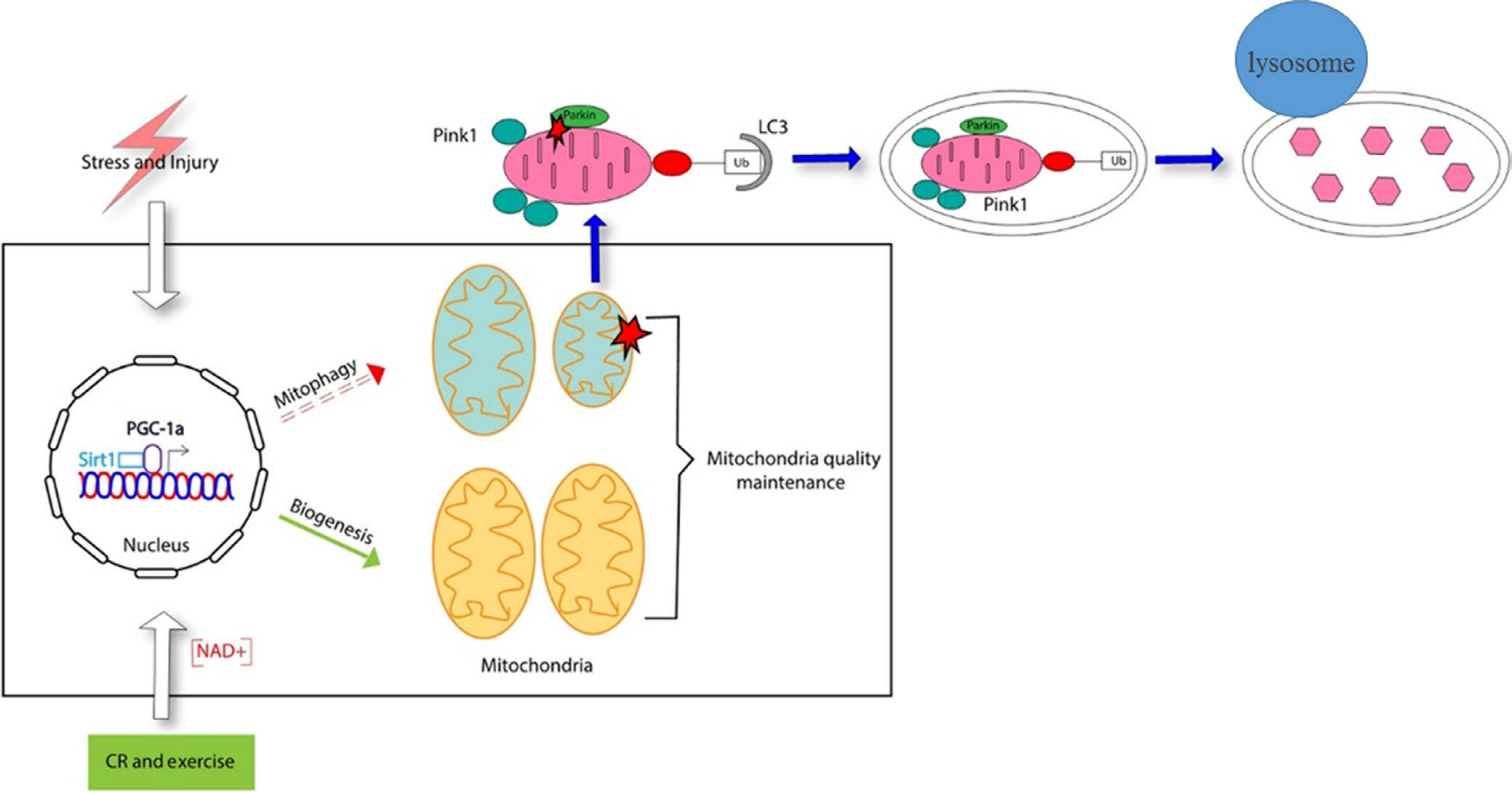
The proteins involved in the process of mitochondrial biogenesis and mitophagy that help to maintain the mitochondria quality. Caloric restriction (CR), physical exercise, and energetic status elevate SIRT1 or NAD^+^ levels that promote the nuclear translocation of PGC‐1α, which would enhance the transcription of genes associated with the mitochondrial function and biogenesis. Supplementing with NAD^+^ also enhance SIRT1 activity and trigger mitophagy

While mitochondria are critical in regulating cellular energy balancem, they also have well‐described roles in the maintenance of essential cellular functions. These include processes such as cellular differentiation, regulation of the cellular growth cycle, and cell death.^2^

## 2 MITOCHONDRIAL BIOGENESIS AND ITS REGULATORS

Mitochondrial biogenesis is regulated to adapt the mitochondrial population to a cell's energy demands in response to the conditions of growth, cell division, and changes in oxidative stimuli and hormones. Mitochondrial biogenesis depends on the coordinated expression of nuclear and mitochondrial DNA. Mitochondrial transcription factor A (TFAM) and nuclear respiratory factors 1 and 2 (NRF1 and NRF2) regulate the biogenesis of mitochondria.^3^, ^4^ The PPARγ coactivator‐1 family of transcription coactivators, sirtuins and AMPK, also are involved in regulating gene expression during mitochondria biogenesis (Figure 2).

### 2.1 PGC‐1 families

There are three members in the PPARγ coactivator‐1 (PGC‐1) family: PGC‐1α, PGC‐1β, and PGC‐1‐related coactivator (PRC). The PGC‐1 coactivators respond to different stimuli to promote mitochondrial gene expression.^5^, ^6^ PGC‐1α, the master regulator of mitochondrial biogenesis, directly interacts with NRF1 to increase the transcription of the genes regulated by NRF1. Overexpression of PGC‐1α in cerebellar neurons increases mitochondrial density by 30% and protects the neurons against mutant synuclein A53T‐α or mutant Huntingtin gene (Htt)‐induced degeneration.^7^ The brains of mice lacking PGC‐1α presents microvacuolation and neuronal loss, which highlights the important role of PGC‐1α in the nervous system.^8^, ^9^ In fact, the common character of several neurodegenerative diseases, including Alzheimer's disease, Parkinson's disease, and Huntington's disease, is the impaired function of PGC‐1α.

PGC‐1β plays a role in the maintenance of basal mitochondrial functions.^7^ PRC, which is expressed ubiquitously, also enhances the gene transcription of NRF1. PRC mainly regulates gene expression in proliferating cells and in growth‐regulated mitochondrial biogenesis.^10^ PRC depletion results in the aggregation of atypical mitochondria and severe respiratory chain dysfunction.^11^

### 2.2 Sirtuins

Sirtuins are class III protein deacetylases that consume one molecule of NAD^+^ during each deacetylation cycle. Evidence form the past research indicates that sirtuins regulate the aging process and extend life span.^12^, ^13^ Sirtuin levels are influenced by diet, exercise, and environmental stress. There are seven mammalian sirtuins, SIRT1‐7. In addition to deacetylating histones or other specific transcription factors to regulate gene expression, the mammalian sirtuins also regulate the activities of metabolic enzymes to response the calorie restriction or other stresses.

SIRT1 is the most studied sirtuin. Research has demonstrated that overexpression of SIRT1 can alleviate diseases, including neurodegenerative diseases, diabetes, and liver steatosis.^14^, ^15^ SIRT1 deacetylates various transcription factors and coactivators, including PGC‐1α, the tumor suppressor p53, and FOXO to enhance the transcription of genes regulated by these factors.^16^ In the energy‐deficient condition caused by disease or injury, SIRT1 activation has neuroprotective effects by promoting mitochondrial biogenesis and triggering the turnover of damaged mitochondria.^17^

Unlike SIRT1, which mostly localizes to the nucleus, SIRT3 localizes to mitochondria and is essential to mediate the response to oxidative stress by activating superoxide dismutase‐2 (SOD2).In response to oxidative stress, SIRT3 deacetylates SOD2 to enhance its ability to scavenge ROS.^18^ Normally, the endogenous SOD2 is inactivated by acetylation and it is important for antioxidant enzymes to counteract cellular ROS. SIRT3 deficiency dramatically exacerbates the degeneration of nigro‐striatal dopaminergic neurons in 1‐methyl‐4‐phenyl‐1,2,3,6‐tetrahydropyridine (MPTP)‐induced Parkinson Disease mice.^19^ Overexpression of SIRT3 decreases the excitotoxicity of *N*‐methyl‐d‐aspartic acid (NMDA) to the cultured mouse cortical neurons.^20^

### 2.3 AMP‐activated protein kinase

AMP‐activated protein kinase (AMPK) is an important regulator of cellular metabolism in eukaryotes. AMPK is activated by the increased AMP/ATP ratio which leads to enhanced glucose transport, fatty acid oxidation, and so on.^21^ AMPK also regulates mitochondria function. On one hand, AMPK can stimulate mitochondria biogenesis through increasing the gene transcription regulated by PGC‐1α; on the other hand, AMPK can acutely trigger the destruction of existing defective mitochondria through Unc‐51‐like autophagy activating kinase (ULK1)‐dependent mitophagy. The dual processes controlled by AMPK have the net effect of replacing existing defective mitochondria with new functional mitochondria. AICAR (5‐aminoimidazole‐4‐carboxamide ribonucleoside), the agonist of AMPK, has neuroprotective effects by reducing β‐amyloid peptide (Aβ) production in neuronal cell culture in the AD cellular model.^22^

## 3 MITOCHONDRIAL ANOMALIES WITH AGING

Aging induces many potentially interconnected defects and is a common risk factor for adult human diseases. During the aging process, multiple mitochondrial anomalies may occur, including bioenergetic deficiency, the increased oxidative stress from respiratory chain, and the accumulation of the dysfunctional mitochondria.^23^, ^24^ Damaged mitochondrial DNA (mtDNA) and the accumulation of injured mitochondria are considered major contributors to aging.

Mutation in mtDNA reduces the lifespan of mice and humans.^27^ According to the mathematical models, there is only a limited clonal expansion of somatic mtDNA mutations that can occur in short‐lived organisms like fruit flies. The experimentally obtained very high mtDNA mutation levels, which are unlikely to be found in nature, also reduce the lifespan of fruit flies. Additionally, adulthood is less sensitive to mtDNA mutations than is embryonic development.^28^

### 3.1 Mitochondrial damage and mutation caused by ROS and replication errors

Impairment of mitochondrial dynamics can result in reduced oxidative phosphorylation and cell death. Mitochondria produce ATP for cellular energy requirements, and ROS are toxic by‐products generated during OXPHOS in mitochondria. Mitochondria generate about 90% of the ROS present in cells. Excess ROS may cause oxidative damage to both nuclear DNA (nDNA) and mtDNA, which may contribute to many age‐related disease states.^29^ Compared to nDNA, mtDNA is prone to be damaged by oxidative stress because it is not protected by associated histones and other chromatin proteins and is near the ROS‐generating respiratory chain.

Besides ROS, replication errors and failure of the repair mechanisms may be the more important reason for accumulation of mtDNA mutations.^30^ mtDNA replication is independent of cellular division, so the replication rate is higher than that of nDNA.^31^ In the meanwhile, there is a decrease in the DNA repair enzymes such as DNA polymerase 1 and endonuclease in aged tissue.^26^ Ultra‐deep sequencing was used to study genome‐wide mtDNA mutation load; the sequence analysis showed that most somatic mtDNA mutations occur as replication errors during development and do not result from damage accumulation in adult life.^32^

### 3.2 Accumulation of dysfunctional mitochondria

Fusion and fission proteins control mitochondrial dynamics. Dysfunctional mitochondria are marked and selectively removed by the specific autophagic process called mitophagy. Through the process of fission, the dysfunctional mitochondria are selected for mitochondrial fragmentation, then turnovered by mitophagy and degraded in lysosomes. Therefore, fission is important for keeping mitochondrial quality and integrity. The level of both autophagy and mitophagy decline with aging,^33^, ^34^ which results in an accumulation of dysfunctional mitochondria, advanced oxidative stress, and increased cell apoptosis. Dysfunctional mitochondrial accumulation occurs in all tissues during aging, including skeletal muscle, liver, and brain.

## 4 MITOCHONDRIAL ABNORMALITIES LEAD TO NEURODEGENERATIVE DISEASES

Mitochondrial dysfunction occurs in most neurodegenerative diseases, including Parkinson's diseases (PD), Alzheimer's (AD), Huntington's disease (HD), Friedreich's ataxia (FRDA), and amyotrophic lateral sclerosis (ALS).

### 4.1 Parkinson's disease

Parkinson's disease is a widespread neurodegenerative disease. The primary hallmark of PD is the loss of dopaminergic neurons of the substantia nigra (SN). Several important genes including PARK7 (encoding DJ‐1), α‐synuclein, parkin, PINK1, or LRRK2 have pathogenic mutations in PD which cause defects in mitochondrial dynamics and function.^36^ Specifically, mutation of α‐synuclein leads to its aggregation and these α‐synuclein aggregates delay fusion of phagosomes with lysosomes during the mitophagic process. Meanwhile, PINK1 deletion results in increased oxidative stress within mitochondria.^37^ In addition, environmental toxins cause mitochondrial dysfunction and are regarded as risk factors for PD as implicated in PD animal or cell models. For example, the complex I inhibitors MPTP, rotenone, pyridaben, and fenpyroximate can mimic the pathological features of PD at low doses and lead to neurodegeneration in flies, rodents, and mammalian cell culture models. Recently, the MitoPark mouse, a model that specifically lacks the gene for TFAM, has become the new genetic model for PD. The mitochondria dysfunction in dopaminergic neurons in MitoPark mice mimics many distinct characteristics of PD, including progressive and selective loss of dopamine neurons, motor deficits, and accumulation of inclusion bodies.^38^

### 4.2 Alzheimer's disease

Alzheimer's disease is a common form of dementia that is associated with aging. The disease‐defining appearance of h Aβ aggregates and Tau pathologies correlate with mitochondrial dysfunctions in neurons. Aβ is generated through the cleavage of Aβ precursor protein^39^ by α‐, β‐, and γ‐secretases. The increased oxidative stress resulting from mitochondrial dysfunction generates the lipid peroxidation product 4‐hydroxynonenal, which covalently modifies the γ‐secretase complex and leads to amplified secretase activity. Increased γ‐secretase activity results in accelerated Aβ accumulation.^40^, ^41^ Hyperphosphorylation of Tau protein (pTau) is another hallmark of AD along with Aβ.^42^ Elevated Ca^2+^ and ROS levels during mitochondrial dysfunction both contribute to the accumulation of pTau aggregates.^43^, ^44^ Melov et al^45^ showed that mitochondrial SOD2 deficiency can result in pTau aggregates in mice, a symptom that is reversible by the administration of antioxidants. Most recently, Sorrentino et al^46^ also demonstrated that amyloid aggregation in cells can be reduced by pharmacologically and genetically targeting the mitophagy process.

### 4.3 Huntington's disease

Huntington's disease is a neurodegenerative disease caused by CAG repeat expansion in the mutant HTT (or IT15) gene, which increases the size of the polyglutamine (polyQ) tract in the *N*‐terminal of the Huntington (Htt) protein. The mutant Htt protein with expanded polyQ forms aggregates. This aggregation recruits other proteins and mitochondria, adversely affecting the mitochondrial fission‐fusion process and disrupting the mitochondrial transportation along axons and dendrites.^47^, ^48^ Mutant Htt may interact directly with the outer mitochondrial membrane and destabilize the membrane while increasing the sensitivity to Ca^2+^ or other apoptotic inducers.^50^ Meanwhile, ROS production also increases in HD patients and the mouse models, causing mitochondrial impairment.^51^ In HD patients and mouse models, mitochondrial fragmentation increases while the motility and respiration decrease. 3‐Nitropropionic acid (3‐NP), an inhibitor of the mitochondrial citric acid cycle, produces the selective striatal degeneration and mimics the progressive locomotor deterioration of HD.^52^, ^53^ Glyceraldehyde 3‐phosphate dehydrogenase (GAPDH) is a key molecule in the glycolytic pathway. In normal cells, oxidized inactive GAPDH (iGAPDH) helps initiate the engulfment of the damaged mitochondrion into the lysosome for degradation. However, Hwang et al^55^ have shown that expanded polyglutamine repeats in HD cell models abnormally interacted with GAPDH, which stalled the GAPDH‐mediated mitophagy.

### 4.4 Friedreich's ataxia

Similar to HD, Friedreich's ataxia (FRDA) is an inherited neurodegenerative disorder caused by homozygous DNA repeat expansion mutation. FRDA's GAA expansion mutation leads to deficiency of the frataxin protein, causing mitochondrial dysfunction through promoting ROS production.^56^ Recently, studies by Abeti et al^57^ revealed that FRDA mouse models show a decrease in mitochondrial membrane potential that is caused by an activity imbalance between Complex I and II in the electron transport chain. This imbalance causes ROS generation in the mitochondrial intermembrane space and the matrix, and the subsequent lipid peroxidation results in neuron degeneration. PGC‐1a, the master regulator of mitochondria biogenesis, also plays a role in FRDA development. Lin et al^58^ demonstrated that in diseased FRDA mouse models, both PGC‐1a and its downstream effectors are significantly reduced compared to healthy controls. This impairment occurred early in the mitochondrial biogenesis pathway and is considered a potential therapeutic target for FRDA treatment.

### 4.5 Amyotrophic lateral sclerosis

Amyotrophic lateral sclerosis is a progressive disease that affects motor neurons in spinal cord and brain. Over 90% of ALS patients are sporadic. The ALS patients have movement abnormalities as well as a progressive loss of intellectual function. Mitophagy, the process of eliminating damaged mitochondria, plays a significant role in the ALS disease mechanisms. The neuromuscular junction (NMJ) of SOD1^G39A^ mice, an ALS disease model, contains significantly fewer phagosomes than do in wild‐type mice, indicating a disruption in mitophagy. The mitophagy‐related proteins PINK1 and Parkin also are downregulated. In PINK1‐Parkin double‐knockout models, mice experience exacerbated NMJ degeneration and axon swelling which corresponds to ALS symptoms.

Furthermore, double‐knockout mice show an increased amount of ATP synthase beta subunit. This suggests that the increasing quantity of mitochondria at the junction resulted from dysfunctional mitophagy.^59^

## 5 THE ELIMINATION OF ABNORMAL MITOCHONDRIA

### 5.1 Mitophagy maintains the quality of mitochondria in cells

Mitophagy is part of the mitochondrial quality control system and is regulated by mitochondria fission‐ and fusion‐promoting proteins. In these processes, impaired mitochondria are engulfed into an autophagosome, which then fuses with a lysosome for degradation by lysosomal enzymes (Figure 2). Mitochondrial dynamics is a crucial process in maintaining proper mitochondrial morphology and in regulating mitochondrial function, responses to apoptotic stimuli, and monitoring mitochondrial quality.^60^, ^61^

The PINK1/Parkin interaction is crucial in regulating mitophagy in mammalian cells. PTEN‐induced putative kinase 1 (PINK1) is a serine/threonine protein kinase present in the cytosol but also targeted to the outer mitochondrial membrane. Mitochondria with positive mitochondrial membrane potential import and degrade PINK1, preventing its accumulation on the outer mitochondrial membrane. PINK1 accumulates on the impaired mitochondria with a decreased Δψm. Parkin, a component of a multi‐protein E3 ubiquitin ligase complex, binds with PINK1 accumulated on the impaired mitochondria and tags the damaged mitochondria with ubiquitin for degradation through mitophagy.^62^, ^63^

In addition, increasing evidence supports, that mitophagy also can occur in a Parkin‐independent way. Without the participation of Parkin, some proteins, such as NIX, FUNDC1, or BNIP3, and cardiolipin directly interact with LC3 protein and engulf the dysfunctional mitochondria into autophagosome.^64^, ^65^ Meanwhile, other E3 ubiquitin ligases such as SMURF1 and MUL1 also can ubiquitinate the damaged mitochondria and promote mitophagy.^67^

Endosomes also play a role in mitochondrial elimination. Dysfunctional mitochondria are marked with ubiquitin by parkin, sequestered into Rab5‐positive early endosomes, and ultimately delivered into lysosomes for degradation. In certain cell types, disruption of the endosomal pathway through loss of Rab5 function increases the likelihood of cell death due to mitochondrial stress.^68^

### 5.2 Abnormal mitochondrial function affects lysosomal activity

Maintaining mitochondrial homeostasis requires the cooperation of mitochondrial biogenesis, mitochondrial fusion, fission, and mitophagy. The lysosome is the main degradation and recycling organelle. Mitophagy tags and delivers the dysfunctional mitochondria to the lysosome for degradation. Meanwhile, mitochondrial dysfunction also affects the structure and function of lysosomes.^69^ Mfn2 promotes lysosomal autophagocytosis. Mfn2 depletion in cardiomyocytes retards the fusion of autophagosomes with lysosomes.^70^ Phagocytosis also can be impaired by the depletion of proteins AIF, OPA1, or PINK1, and by chemical inhibition of the electron transport chain, causing the enlarged lysosomal vacuoles. CCCP increases mitochondrial ROS levels via membrane depolarization and specifically activates the lysosomal TRPML1 channels, causing lysosomal Ca^2+^ release. This activation triggers nuclear translocation of transcription factor EB (TFEB). This calcineurin‐dependent transcription factor can activate the autophagy/lysosome pathway by regulating the biogenesis of autophagy/lysosome organelles.^71^

## 6 METHODS TO ENHANCE MITOCHONDRIAL FUNCTION

### 6.1 Pharmacological methods

There are several pharmacological strategies aimed at triggering mitochondria biogenesis to treat neurodegenerative diseases. Rosiglitazone and bezafibrate can activate the PPAR‐PGC‐1α axis, and the SIRT1 agonists quercetin and resveratrol can activate sirtuins and AMPK.^72^, ^73^ Bezafibrate increase the mitochondrial proteins and mitochondrial ATP generating capacity; as the result, it had a neuroprotective effect in this mouse model of mitochondrial encephalopathy.^76^ Rosiglitazone can increase mitochondrial mass and attenuate mitochondrial dysfunction in mutant Htt‐expressing cells.^77^

Several antioxidants such as creatine, coenzyme Q10, and mitochondrially‐targeted antioxidants/peptides seems to improve the patient's ankle mobility in PD clinical trials.^78^ However, most of the antioxidants, such as dimebon, seem to have beneficial effects in the AD preclinical research, but failed in the AD clinical trials.^79^, ^80^ Since mitochondrial dysfunction occurs at the early stage of diseases, the method of pharmacological interventions should be considered.

Besides enhancing mitochondria biogenesis and the scavenging of ROS, inducing mitophagy would be another strategy to maintain mitochondria homeostasis. Urolithin A, a metabolite of ellagitannins produced by the human gut microbiota, induces mitophagy, prevents the accumulation of dysfunctional mitochondria with age, and prolongs lifespan in *Caenorhabditis elegans* and increases muscle function in rodents.^81^ Urolithin A can go through the blood‐brain barrier, which may have neuroprotective effects against neurodegenerative diseases. Spermidine is produced from putrescine and could be a precursor of spermine generation. Spermidine acts as acetylase inhibitor and induces autophagy in a SIRT1‐independent manner.^82^ Therefore, spermidine promotes basal autophagic flux and stimulates mitophagy repairing mitochondrial activity in aged cardiomyocytes.^83^ Spermidine feeding protects from age‐induced memory impairment in an autophagy‐dependent manner.^84^ Spermidine induces the formation of mitophagosomes and decreases the aggregation of dysfunctional mitochondrial through the PINK1/Parkin pathway.^85^

### 6.2 Caloric restriction

Caloric restriction (CR) is to decrease the calories intake but maintain all the essential nutrients and without malnutrition. CR decreases the production of ROS and reduces oxidative DNA damage, slows down the transcriptional changes associated with aging.^86^

Sirtuins are considered to have an important role in mediating the beneficial effects of CR on longevity.^87^, ^88^ Similar with CR, SIRT1 overexpression is helpful to extend the life span and decrease the disease syndromes of neurodegenerative diseases. CR induced the expressions of sirtuins, such as SIRT1, SIRT3, SIRT5, and SIRT7.^89^ SIRT1 knockout mice cannot live longer even with the CR diet.^90^ CR delayed the onset of prion disease mice but failed to delay the onset in the SIRT1 knockout strain.^91^ Knocking out the mitochondrial SIRT3 prevents the protective effect of CR against hearing loss.^92^

Caloric restriction enhances the number of mitochondrial cristae as well as the number of mitochondria in per cell.^93^ CR also prevents excitotoxic conditions through the indirect decrease in mitochondrial permeability and calcium retention. These are mediated through CR‐activated SIRT3 deacetylation and inhibition of cyclophilin D, a peptidylprolyl isomerase.^94^

Additionally, CR inhibits the PI3K/AKT pathway, induces autophagy, which may increase mitophagy and maintain mitochondria homeostasis.

### 6.3 Physical exercise

Some research results show that exercise might be helpful in retarding the progress of neurodegenerative diseases,^95^, ^96^ which may be associated with the recovery of mitochondrial function by exercise. Exercise stimulates brain mitochondrial activity. Exercise not only increases resistance against rotenone, an inhibitor of complex I activity, but also increases mRNA expression of TFAM and Ndufa6, subunits of mitochondrial complex I.^97^ At the same time, exercise increases mtDNA repair capacity in the mouse hippocampus and activates mitochondrial uncoupling proteins (UCP) which can regulate mitochondrial proliferation^98^ and control the production of mitochondrial‐derived ROS. Exercise upregulates UCP2 levels in the hippocampus, lowers cellular oxidative stress^99^ and can activate autophagy, which is helpful in maintaining muscle mass.^100^, ^101^ We have found that exercise could ameliorate the detrimental effect of chloroquine on skeletal muscles through restoring autophagic flux^104^ and activating the autophagy/lysosomal pathway through AMPK pathway in cerebral cortex and striatum.^105^ The enhanced mitophagy lessens the level of dysfunctional mitochondria to maintain a high quality of mitochondria in cells.

## 7 CONCLUDING REMARKS

Mitochondrial dysfunction, the downstream oxidative stress and impaired autophagy/lysosomal activity are the main factors involved in neurodegeneration. Thus, drugs that improve mitochondria function, scavenge the excessive ROS, or enhance the autophagic flux may have the potential to treat neurodegenerative diseases. However, pharmacological agents enhancing mitochondria integrity to treat neurodegenerative diseases remain to be developed. At this point, both CR and exercise, which can enhance mitochondria biogenesis and the autophagy/lysosome pathway (including mitophagy), maybe helpful in retarding the onset and progression of neurodegenerative diseases.
